# Weighted gene co-expression network analysis unveils gene networks associated with the Fusarium head blight resistance in tetraploid wheat

**DOI:** 10.1186/s12864-019-6161-8

**Published:** 2019-12-03

**Authors:** Ehsan Sari, Adrian L. Cabral, Brittany Polley, Yifang Tan, Emma Hsueh, David J. Konkin, Ron E. Knox, Yuefeng Ruan, Pierre R. Fobert

**Affiliations:** 1 Aquatic and Crop Resource Development Centre, National Research Council Canada, Saskatoon, SK Canada; 2 Swift Current Research and Development Centre, Agriculture and Agri-Food Canada, Swift Current, SK Canada

**Keywords:** *Fusarium graminearum*, Transcriptome profiling, Weighted gene co-expression network analysis, FHB resistance QTL, Tetraploid wheat, Constitutive defense, Plant height, Maturity, SNP discovery

## Abstract

**Background:**

Fusarium head blight (FHB) resistance in the durum wheat breeding gene pool is rarely reported. *Triticum turgidum ssp. carthlicum* line Blackbird is a tetraploid relative of durum wheat that offers partial FHB resistance. Resistance QTL were identified for the durum wheat cv. Strongfield × Blackbird population on chromosomes 1A, 2A, 2B, 3A, 6A, 6B and 7B in a previous study. The objective of this study was to identify the defense mechanisms underlying the resistance of Blackbird and report candidate regulator defense genes and single nucleotide polymorphism (SNP) markers within these genes for high-resolution mapping of resistance QTL reported for the durum wheat cv. Strongfield/Blackbird population.

**Results:**

Gene network analysis identified five networks significantly (*P* < 0.05) associated with the resistance to FHB spread (Type II FHB resistance) one of which showed significant correlation with both plant height and relative maturity traits. Two gene networks showed subtle differences between *Fusarium graminearum*-inoculated and mock-inoculated plants, supporting their involvement in constitutive defense. The candidate regulator genes have been implicated in various layers of plant defense including pathogen recognition (mainly Nucleotide-binding Leucine-rich Repeat proteins), signaling pathways including the abscisic acid and mitogen activated protein (MAP) kinase, and downstream defense genes activation including transcription factors (mostly with dual roles in defense and development), and cell death regulator and cell wall reinforcement genes. The expression of five candidate genes measured by quantitative real-time PCR was correlated with that of RNA-seq, corroborating the technical and analytical accuracy of RNA-sequencing.

**Conclusions:**

Gene network analysis allowed identification of candidate regulator genes and genes associated with constitutive resistance, those that will not be detected using traditional differential expression analysis. This study also shed light on the association of developmental traits with FHB resistance and partially explained the co-localization of FHB resistance with plant height and maturity QTL reported in several previous studies. It also allowed the identification of candidate hub genes within the interval of three previously reported FHB resistance QTL for the Strongfield/Blackbird population and associated SNPs for future high resolution mapping studies.

## Background

Durum wheat (*Triticum turgidum* L. ssp. *durum* (Desf.) Husn.) is one of the major cereal food crops grown in the temperate regions of the world. The sustainability of durum wheat production is threatened by the yield and quality losses caused by Fusarium head blight disease (FHB). The dominant causal agent in Canada, *Fusarium graminearum* Schwabe, produces mycotoxins such as deoxynivalenol (DON) [1, 2] and kernels contaminated with DON are not suitable for human consumption. The yield and quality losses can be alleviated by integrated management practices such as crop rotation, crop residue management, fungicide application and growing FHB resistant varieties. Due to limitations associated with fungicide application, including costs and the development of fungicide resistance in the pathogen population, breeding wheat varieties with high levels of resistance is the most desirable method of control.

Dissecting the genetics of resistance to FHB has been confounded by the polygenic nature of resistance, requiring a quantitative approach for evaluation and analysis. Several quantitative trait loci (QTL) conferring resistance to initial infection or incidence (Type I resistance) and spread or severity (Type II resistance) have been identified in hexaploid wheat [3]. Type I resistance is usually associated with morphological traits such as plant height, flowering time, awn morphology and anther retention [4]. However, Type II FHB resistance is associated with transmission of systemic defense signals to non-infected spikelets, which inhibits the spread of the fungus to the adjacent rachis tissues [5, 6].

Fewer sources of FHB resistance have been reported in durum wheat and most durum wheat varieties are susceptible or moderately susceptible to FHB [3, 7]. Characterization of novel resistance sources in durum wheat and its tetraploid relatives is required for improving the levels of genetic resistance. Moderate resistance to FHB has been previously reported from tetraploid relatives of durum wheat such as *T. turgidum* ssp. *dicoccoides* [8], *T. turgidum* ssp. *dicoccum* [7, 9] and *T. turgidum* ssp. *carthlicum* [7, 10].

To date, only candidate FHB resistance genes associated with an FHB resistance QTL on chromosome 3BS present in line Sumai 3 (*Fhb1*) has been identified [11]. One of the candidate FHB resistance gene within the *Fhb1* interval encodes a pore-forming toxin-like protein containing a chimeric lectin with two agglutinin domains and one ETX/MTX2 toxin domain. Recently, Su et al. [12] identified another candidate FHB resistance gene within the *Fhb1* interval encoding a putative histidine-rich calcium-binding protein. The *Fhb1* locus also confers resistance to DON accumulation through conversion of DON to a less toxic conjugate DON 3-glucoside [13]. The DON-degrading activity in lines carrying the *Fhb1* locus has been associated with uridine diphosphate (UDP)-glycosyltransferase activity [13]; however, genes with UDP-glycosyltransferase activity are not present within the *Fhb1* QTL interval [14]. The availability of multiple candidate resistance genes in the *Fhb1* QTL interval [15] supports the complex genetic architecture of this locus.

Candidate resistance genes have been identified for *Qfhs.ifa-5A*, a FHB resistance QTL on chromosome 5AL mediating Type I resistance [16] and *Fhb2*, on chromosome 6BS, mediating Type II FHB resistance [17], both present in line Sumai 3, and a resistance QTL on chromosome 2DL present in cv. Wuhan-1 [18]. Additional research is required to confirm the resistance gene(s) associated with these QTL. Despite similarity between the loci conferring FHB resistance in tetraploid and hexaploid wheat [9, 10, 19], none of FHB resistance QTL reported in tetraploid wheat has been resolved to the gene level.

*Fusarium graminearum* is a hemibiotrophic plant pathogen. Initial disease symptoms appear 48 h post infection, concurrent with a switch from a non-symptomatic sub-cuticular and intercellular growth to a intracellular necrotrophic phase [20]. A previous study indicated that the pathogen hijacks host signaling for the switch to the necrotrophic phase [21]. Partial resistance is often achieved through reducing the spread of fungus inside the spike and rachis tissues [22, 23]. Studying the components of plant defense conferring lower colonization of the wheat spike is a key step toward the discovery of FHB resistance mechanisms and hence the identification of novel strategies for improving resistance to FHB.

The interaction of wheat with *F. graminearum* has been intensively studied during the past decade [24]. These studies mostly consisted of comparisons of transcriptomic profiles from FHB resistant and susceptible lines. The throughput and the precision of these studies have been largely improved by the advent of next generation RNA-sequencing technology and the release of the wheat reference genome [25]. Several mechanisms of FHB resistance were proposed such as stronger and faster expression of defense responses in more resistant versus more susceptible lines [26] and subverting the virulence mechanisms of the pathogen by the activities of genes such as ABC transporters, UDP-glucosyltransferase and proteinase inhibitors [27]. A blend of phytohormone signaling pathways is induced upon the infection of wheat by *F. graminearum*, with the contribution of each to resistance varying depending on genotype and the pathogen isolate [24]. The biosynthesis of these phytohormones are altered by an intricate network of cross-talk allowing the lines with resistance to respond to infection in a timely fashion [24]. Both negative and positive involvement of the ethylene (ETH) signaling pathway in FHB resistance was proposed [22, 28, 29]. The sequential expression of the salicylic acid (SA) and jasmonic acid (JA) signaling pathways in the resistant line Wangshuibai suggested the involvement of these hormones in resistance [30]. The activation of the SA signaling pathway was delayed in a FHB susceptible line derived from a Wangshuibai mutant, corroborating the association of resistance with the timing of the SA signaling. Priming resistance to FHB through inoculation of wheat spikes with a *F. graminearum* isolate impaired in DON production was associated with the induction of the ETH, JA and gibberellic acid (GA) signaling pathways [31]. The GA signaling pathway regulates plant height, which is often negatively associated with FHB severity [32, 33]. The theory that FHB resistance is passively modulated by plant height is changing with the emerging evidence of the involvement of the GA signaling pathway in FHB resistance [31, 34]. The abscisic acid (ABA) and GA signaling antagonistically modulate FHB resistance in hexaploid wheat, supporting the importance of the ABA and GA cross-talk in the outcome of the wheat-*F. graminearum* interaction [35]. As a virulence mechanism, *F. graminearum* is equipped with pathogenic effectors that interfere with these signaling pathways [36].

A variety of down-stream defense responses is induced by *F. graminearum* infection for example chitin binding proteins, chitinases, glucanases and thaumatin-like proteins [37–40]. The cereal cysteine-rich proteins such as defensin, thionin, nonspecific lipid transfer proteins, puroindoline, hevein and knottin also show antifungal activities against *F. graminearum* [41, 42]. The pore-forming proteins have antifungal activities against *F. culmorum* in vitro [43] and one of the FHB resistance gene identified thus far encodes a member of this protein family [11]. The down-stream defense responses also include the inhibitors of the pathogen cell wall degrading enzymes such as polygalactronases and xylanases [44, 45]. In addition, wheat responds to *F. graminearum* infection by reinforcing the cell wall at the site of penetration attempts by papillae formation and by fortifying the cell wall through lignin deposition [22, 46, 47]. FHB resistant lines have been shown to accumulate higher concentration of *p*-coumaric acid in the infected spikelet tissues [48]. *P*-coumaric acid is a precursor of phenolic compounds synthesized in phenylpropanoid pathway [48].

Despite intensive research on FHB resistance mechanisms, the constitutive aspect of FHB resistance in wheat is poorly understood. Constitutive resistance to FHB is attributed to anatomical differences between the susceptible and resistance genotypes [49] and preformed physical barriers, such as phenolic compounds deposited in the cuticular wax and in the primary cell wall, that lower the colonization of wheat spikes [50]. For example, Lionetti et al. [50] showed that cell wall composition varied between FHB resistant lines derived from line Sumai 3 and the susceptible durum wheat cv. Saragolla in lignin monolignols, arabinoxylan substitutions and pectin methylesterification. In addition, *TaLTP3*, a candidate resistance gene in the interval of the *Qfhs.ifa-5A* QTL encoding a lipid transfer protein, showed higher levels of basal expression in the resistant line Sumai 3 [51]. Similarly, near isogenic lines (NILs) carrying resistance alleles showed higher levels of basal expression of seven candidate resistance genes associated with the FHB resistance QTL on chromosome 2D present in cv. Wuhan-1 compared to lines with susceptible alleles [18].

The FHB resistance of a doubled haploid (DH) population from a cross between durum wheat cv. Strongfield and *T. turgidum* ssp. *carthlicum* line Blackbird was previously evaluated in greenhouse trials, and field nurseries over several years and locations [10, 19]. FHB resistance QTL were reported on chromosomes 1A, 2A, 2B, 3A, 6A, 6B and 7B with the resistance allele belonging to Blackbird for the QTL on chromosomes 1A, 2A, 3A and 6B. These studies paved the way for utilization of Blackbird resistance in the breeding program; understanding the mechanism of resistance conferred by each QTL is required for their more effective utilization in breeding programs. Understanding the molecular defense responses associated with these QTL allows the identification of FHB resistance candidate genes and the development of gene-based diagnostic markers desired for marker-assisted selection (MAS).

In this study, a weighted gene co-expression network analysis was applied to identify gene networks associated with the reaction to *F. graminearum* in Blackbird, cv. Strongfield and two DH lines of the cv. Strongfield/Blackbird mapping population with extreme resistance and susceptible phenotypes. The analysis allowed the identification of five gene networks significantly associated with FHB resistance as well as genes with the highest network connectivity (hub genes) within each network having potential regulator functions. The possible contribution of the hub genes to FHB resistance especially those lying within the interval of the reported FHB resistance QTL in the cv. Strongfield/Blackbird population is discussed. Single nucleotide polymorphism (SNP) within the hub genes were identified for future high-resolution mapping studies.

## Methods

### Plant materials

The tetraploid wheat lines used for this study include *T. turgidum* ssp. *durum* cv. Strongfield (SF), *T. turgidum* ssp. *carthlicum* line Blackbird (BB), one transgressive resistant (R) and one transgressive susceptible (S) DH line of the SF/BB population carrying alternative alleles at the reported FHB resistance QTL on chromosomes 1A, 2B, 3A and 6B [19]. Strongfield (AC Avonlea//Kyle/Nile) is a spring durum wheat cultivar adapted to the semi-arid environment of the northern Great Plains developed at the Swift Current Research and Development Centre (SCRDC) of Agriculture and Agri-Food Canada (AAFC). Blackbird was a selection out of *T. turgidum* ssp. *carthlicum* line REB6842, which was obtained from Dr. Maxim Trottet of INRA Centre de Recherches de Rennes, in France [52] and has been used as an exotic source of FHB resistance in the SCRDC breeding program. Plants (one per each pot) were grown in 10 cm diameter round pots containing a soilless mixture of Sunshine Mix No. 8 (Sun Grow Horticulture® Ltd., Vancouver, Canada) in a growth cabinet with average daily temperate of 23.5 °C under a 18/6 h light/dark regime supplied from florescent lighting. The experiment was conducted as a randomized complete block design with three replicates.

### Fungal inoculation

An aggressive 3-acetyl-deoxynivalenol (3ADON) producing isolate of *F. graminearum* (M9-4-6) collected from Manitoba, Canada and provided by Dr. Jeannie Gilbert at Agriculture and Agri-Food Canada, Cereal Research Centre, Winnipeg, MB was used for inoculation. The fungal isolate was preserved as a spore suspension from a monoconidial culture in a cryopreservation solution containing 10% skim milk and 20% glycerol at − 80 °C. For inoculum preparation, conidia were revitalized on Potato Dextrose Agar medium plates for 8 d at room temperature. Plugs of the fungus taken from the actively growing edge of the colonies were placed in 250 ml Erlenmeyer flasks containing 100 ml of Carboxymethyl cellulose liquid medium [53] and incubated on a rotary shaker for 4 d at room temperature. Conidia were harvested from the culture medium by filtering through 2 layers of cheesecloth and centrifuging the filtrate at 3000 rpm for 5 min. The concentration of suspension was adjusted to 5 × 10^4^ conidia ml^− 1^ using a hemocytometer. The 12 florets (six on opposite sides of the spike) of the top 2/3 portion of the spike were inoculated at 50% anthesis between the lemma and palea of each floret either by injecting 10 μl of conidia suspension for inoculated plants or sterile distilled water for mock inoculated plants. The heads were then sprayed with sterile distilled water and covered with polyethylene transparent plastic bags to maintain high humidity.

### Illumina RNA sequencing

A single head per each inoculated and mock-inoculated plant was collected at 48 h post inoculation and flash frozen in liquid nitrogen. The head tissues were ground to fine powder in an RNAse-free mortar precooled with liquid nitrogen. The RNA from the rachis was processed separately from the palea and lemma and they were pooled in 1:1 ratio for RNA-sequencing. RNA was extracted using Qiagen RNeasy Kit (Qiagen, Hilden, Germany) following the manufacturer’s protocol. The purity of RNA was tested using a NanoDrop ND8000 (Thermo Scientific, Wilmington, USA) and samples with an A260/280 ratio less than 2.0 were discarded. The quantity of RNA was determined using a Qubit® 2.0 Fluorometer (Grand Island, NY, USA) and a Qubit™ RNA broad range assay kit (Invitrogen, Carlsbad, USA) following the manufacturer’s protocol. The integrity of RNA was determined using an Agilent 2100 Bioanalyzer using Agilent RNA 6000 Nano Kit (Agilent Technologies Inc., Santa Clara, USA).

Total RNA (~ 1 μg) for each sample was used for library preparation using Illumina TruSeq® RNA sample preparation v. 2 kit (Illumina, San Diego, USA). The samples were sequenced (2 × 125 cycles, paired-end reads) on the HiSeq 2500 (Illumina, San Diego, USA) using the TruSeq SBS v3-HS 200 cycles Kit (Illumina, San Diego, USA).

### Weighted gene co-expression network analysis

The short reads were filtered to retain only those with a Phred quality score of greater than 20 and a length of at least 60 nucleotides using Trimmomatic v0.36 software [54]. The retained short reads were deposited in the Sequence Read Archive (SRA) of the National Center for Biotechnology Information (NCBI) under BioProject accession PRJNA531693. A total of 563 million filtered short reads were mapped to the International Wheat Genome Sequencing Consortium (IWGSC) hexaploid wheat (Chinese Spring) RefSeq v1.0 [25] using short reads mapper STAR v.2.5.4b [55] following the StringTie v1.3.4b pipeline [56, 57]. Raw reads count per gene were obtained with software htseq-count v0.9.0cp27m [58] and normalized read counts were reported using the relative log expression method available in DESeq2 v1.18.1 [59]. Genes with consistently low expression in more than half of the samples (normalized read counts < 10), and coefficient of variation < 0.4 were filtered out. Normalized read count were subjected to pseudocount transformation using log_2_ eq. (normalized count+ 1). Hierarchical clustering of samples using hclust package of R v3.4.3 [60] supported high correlation among the biological replicates of each treatment, except for one rep of inoculated SF samples which was excluded from analysis (Additional file 1). The remaining 27,284 genes and 23 samples were used for the identification of gene co-expression networks (module) using the Weighted Gene Correlation Network Analysis (WGCNA) software [61]. The model was fit to a power law distribution (network type signed; power = 10), and the genes were clustered using the Topological Overlap Matrix [61] method using the cutree dynamic option (minClusterSize = 50; deepSplit = 2; pamRespectsDendro = FALSE, merging close modules at 0.9). The eigengenes of the modules (ME) and their correlation with FHB Type II rating generated previously by Somers et al. [10] were determined. Genes with the top 10% intramodular connectivity in the modules significantly correlated with Type II FHB resistance were reported as candidate hub genes. To account for the association of FHB severity with plant height and maturity, the correlation of MEs with plant height and maturity data collected by Sari et al. [19] under field condition was also assessed. Plant height was measured on a representative plant from the soil surface to the tip of spikes excluding the awns. Relative maturity was rated using a 1–6 scale (1 = earliest and 6 latest maturity) when 80% or more of the plots had yellow heads, by pinching the seeds and comparing their moisture levels with the parents.

The gene functional annotation was either extracted from the IWGSC RefSeq v1.0 annotation or by reciprocal blast search against the TrEMBL protein database [62]. Clustering of functional annotation of genes belonging to modules significantly correlated with Type II FHB resistance was conducted using Database for Annotation, Visualization and Integrated Discovery (DAVID) v6.2 [63] using *Arabidopsis thaliana* genome as default gene population background and medium classification stringency. The Benjamini adjusted *P* threshold of 0.05 was used to identify significantly enriched clusters. Candidate defense genes in the modules correlated with Type II FHB resistance were identified based on the functional annotation assigned by DAVID and published genes associated with plant defense.

### Assessing the expression of selected candidate hub defense genes with quantitative real time PCR (qRT-PCR)

To confirm the RNA sequencing results, the expression of a single hub gene per five modules identified from WGCNA analysis was assessed using qRT-PCR. Primers were designed based on specificity scores as ranked by Thermoalign software [64] using the first transcript of each gene from the IWGSC RefSeq v1.0 annotations (Additional file 2). Total RNA (~ 1 μg) was used for reverse transcriptase-dependent first strand cDNA synthesis using the high capacity RNA to cDNA kit™ (Applied Biosystems, Warrington, UK) following the manufacturer’s protocol. PCR amplifications were conducted in an ABI StepOnePlus™ Real-Time PCR machine (Applied Biosystems, Foster City, USA) in a 15.5 μl reaction containing 7.1 μl of Applied Biosystems® Fast SYBR® Green Master Mix (Applied Biosystems, Warrington, UK), 0.2 μM of each primer and 5 μl of 1:5 diluted cDNA. The amplification conditions were 95 °C for 3 min, 40 cycles of 95 °C for 10 s, 64 °C for 30 s followed by a melting curve from 60 °C to 95 °C with 0.3 °C intervals. PCR reactions were conducted in triplicate and repeated if the standard deviation of the replicates was higher than 0.2.

Amplification efficiency was calculated for each primer pair and genotype using cDNA stock serially diluted 1:4 (V/V) four times. Dilutions were used for qRT-PCR following the protocol described above. A linear equation was fitted to the cycle of threshold (Ct) values obtained for various cDNA dilutions. Percentile of amplification efficiency (E) was calculated from the slope of the regression line using the eq. E = 10 ^(− 1/slope)^ -1. New primer pairs were designed if E was lower than 99%.

QRT-PCR data were normalized using the α-*tubulin* (TraesCS4A02G065700) as a reference gene using primer pairs designed by Paolacci et al. [65]*.* Expression level was reported as expression fold change relative to mock inoculated samples following the method of Livak and Schmittgen [66]. To be able to compare the gene expression of qRT-PCR and RNA sequencing, the expression ratio from RNA sequencing was calculated from the normalized read counts generated by DESeq2 by dividing that of inoculated with the average of mock-inoculated samples of each genotype. Spearman’s correlation analysis was conducted between expression fold change data of qRT-PCR analysis and expression ratio of RNA-seq analysis using PROC CORR of the Statistical Analysis System (SAS) v9.3 (SAS Institute Inc., Cary, USA).

### Discovery and annotation of the genetic variants within the candidate defense hub genes

The short reads generated for two parental lines SF and BB were combined into two fastq files and were mapped to the IWGSC RefSeq v1.0 assembly using STAR software as described above. The polymorphism among the sequences was called using samtools v1.7 [67] and freebayes v1.1.0 [68]. The resulting variant call format (vcf) file was filtered for mapping quality (QUAL> 40), for mean mapping quality alternate alleles (MQM > 20) and for read depth (total DP > 30). Functional annotation of variants was conducted with SnpEff v4.3 [69] using the annotation of the IWGSC RefSeq v1.0 assembly.

## Results and discussions

### Module construction and module trait-association

**Fig. 1:**
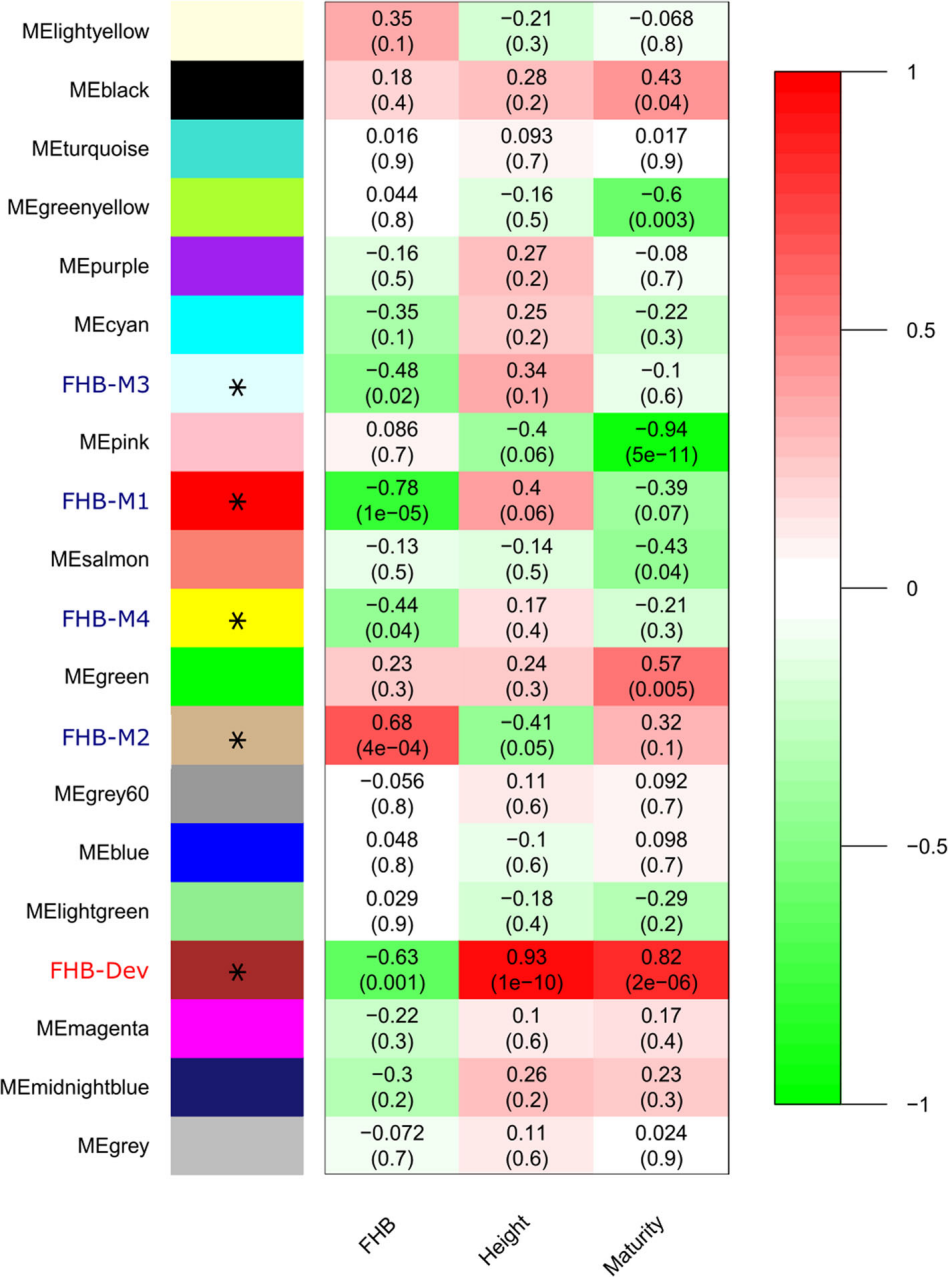
Correlation of module eigengenes (ME) with Type II Fusarium head blight resistance (FHB), plant height (Height) and relative maturity (Maturity) traits. The heat map shows the range of correlation by a color spectrum ranging from green (negative correlation) to red (positive correlation). Numbers in the cells show the correlation coefficient (*r*^*2*^*)* and the correlation probability (*P)* value is denoted in parenthesis. Modules marked with asterisks and named as FHB-M1–4 are significantly (*P* < 0.05) correlated with Type II FHB resistance and that with an asterisk and FHB-Dev is significantly correlated with Type II FHB resistance, Height and Maturity

WGCNA analysis enabled the grouping of genes into 19 co-expression networks (modules) with 350 genes that could not be assigned (assigned to the gray module by default, Fig. 1). Correlation analysis of ME with Type II FHB resistance identified five modules with significant (*P* < 0.05) correlation assigned as FHB-M1, FHB-M2, FHB-M3, FHB-M4 and FHB-Dev. The ME of the FHB-M1 module had the highest correlation with Type II FHB resistance (*r*^*2*^ = − 0.78), followed by the FHB-M2 (*r*^*2*^ = 0.68), FHB-Dev (*r*^*2*^ = − 0.63), FHB-M3 (*r*^*2*^ = − 0.48) and FHB-M4 (*r*^*2*^ = − 0.44) modules. The ME of the FHB-Dev modules had significant correlation with plant height and relative maturity, suggesting the presence of genes with functions in FHB resistance, plant height and maturity within these modules. The correlation of the FHB-Dev ME with plant height and relative maturity was higher than that with Type II FHB resistance.

While studying the genetics of FHB resistance in the SF/BB population, Sari et al. [19] identified FHB resistance QTL co-located with plant height QTL on chromosomes 2A and 3A and with relative maturity QTL on chromosomes 1A and 7B, supporting the association of FHB resistance QTL with plant height and maturity traits. This association had been interpreted as the contribution of plant height and maturity to disease escape in a previous study [70]. The contrasting correlation of the FHB-Dev MEs with FHB resistance (*r*^*2*^ = − 0.63) vs. plant height (*r*^*2*^ = 0.93) in the present study corroborate the negative association of FHB severity with plant height as previously reported [70]. However, the association cannot be solely related to disease escape since spikes were point-inoculated at the optimum infection stage (50% anthesis). A recent study suggested the involvement of the GA signaling pathway in resistance of wheat to FHB, lending support to the physiological effects of plant height genes on resistance to FHB [34]. Interestingly, not all the modules associated with the plant height and relative maturity were correlated with Type II FHB resistance, as an example, the ME of the pink module was highly correlated (*r*^*2*^ = − 0.94) with relative maturity, but was not significantly correlated with FHB resistance.

### Differential expression of eigengenes from modules correlated with FHB resistance among genotypes

**Fig. 2:**
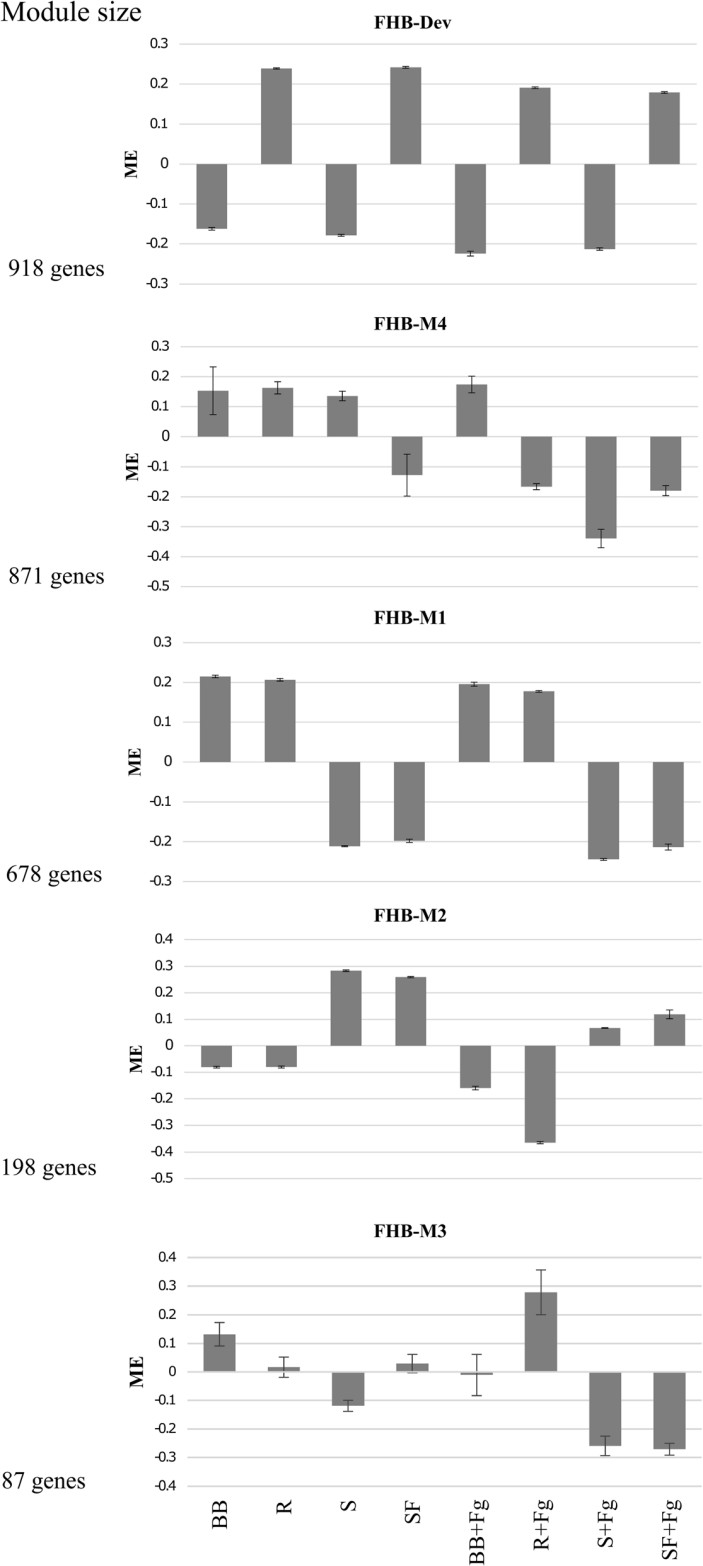
The size (number of genes) and module eigengenes (ME) expression of gene networks correlated with Type II FHB resistance. Genotypes are cv. Strongfield (SF), Blackbird (BB), a transgressive resistant (R) and a transgressive susceptible (S) doubled haploid line from the SF/BB population. Samples were mock-inoculated with water or inoculated with a *Fusarium graminearum* conidial suspension (+Fg). Error bars indicate standard deviations of the mean of three biological replicates

The size (number of genes per module) and ME expression of the five modules significantly correlated with FHB resistance are presented in Fig. 2. The module size varied from 918 to 87 genes with the FHB-Dev module being the largest and the FHB-M3 module the smallest. Expression of the ME for the FHB-Dev and FHB-M1 modules was different among genotypes but was similar between inoculated and mock-inoculated samples of the same genotype. This suggests that genes in these modules may be involved in constitutive defense mechanisms, those not being affected by the pathogen infection. The association of constitutive defense with resistance to FHB was previously proposed [18, 50, 51]. For example, the difference in resistance of durum and bread wheat to FHB was linked with the difference in lignin monolignols composition, arabinoxylan (AX) substitutions and pectin methylesterification of cell wall [50] and resistance was suggested to be linked with the higher basal levels of SA in line Sumai 3 [22]. Most previous transcriptome analyses of wheat-*F. graminearum* interactions focused on differential gene expression analysis after pathogen challenge [24] wherein constitutive defense mechanisms were overlooked. In the present study, the application of gene co-expression network analysis allowed identification of candidate defense genes involved in constitutive defense. The notion that the FHB-M1 module had the highest correlation with FHB resistance suggests that the contributions of constitutive defenses genes in this module might outweigh induced defense mechanisms in the tetraploid wheat germplasm analyzed.

The ME expression of R plants was similar to BB in the FHB-M1 and FHB-M2 modules (Fig. 2), while ME expression of S plants was similar to SF, consistent with inheritance of resistance components from BB and susceptibility from SF. The opposite pattern was observed in the FHB-Dev module, inferring that SF might have contributed to the resistance levels of R plants through the expression of some FHB-Dev module genes. Further support for the contribution of SF alleles to resistance is lent by the report of a Type II FHB resistance QTL on chromosome 2B with the resistance allele derived from SF in the previous studies [10, 19]. Mapping analysis suggested that R carries resistance alleles of both the 1A (derived from BB) and the 2B (derived from SF) FHB resistance QTL [19], which could additively contribute to the higher level of resistance in R than BB.

The FHB-M4 module ME had contrasting expression in inoculated SF and BB plants with R and S plants being more similar to SF than BB (Fig. 2). Since the FHB-M4 module ME is similarly expressed in S and SF, the resistance of BB might be linked to the lower expression of susceptibility genes of the this module. The hierarchical clustering of genotypes based on the expression of whole transcriptome used for WGCNA analysis (Additional file 1) was reminiscent of the FHB-M4 ME expression, as inoculated BB plants formed a distinct cluster that was more related to the mock-inoculated than inoculated plants. Since BB has several undesirable agronomic traits, we considered other traits such as lodging, plant height and maturity for selecting R as the most adapted FHB resistance progeny of the SF/BB population. This may also explain the similarity between the R and SF in the expression of the FHB-M4 module ME.

The expression of the FHB-M2, FHB-M3 and FHB-M4 MEs was largely different in mock-inoculated and inoculated genotypes, suggesting that they carry genes involved in inducible defense (Fig. 2). Knowing the quantitative nature of FHB resistance, the cumulative effect of constitutive and inducible defense mechanisms could theoretically fortify resistance to FHB. FHB-M2 ME expression was different in inoculated BB and R plants. It is likely that genes of the FHB-M2 module contribute to the transgressive expression of resistance in R. Similar to FHB-M4 module, all genotypes but BB showed different ME expression of FHB-M3 module in the inoculated and mock-inoculated samples. The difference between R and other genotypes in the expression of FHB-M3 MEs supports the contribution of this module to transgressive expression of resistance in R.

### Clustering functional annotation of genes belonging to modules significantly correlated with FHB resistance

**Fig. 3:**
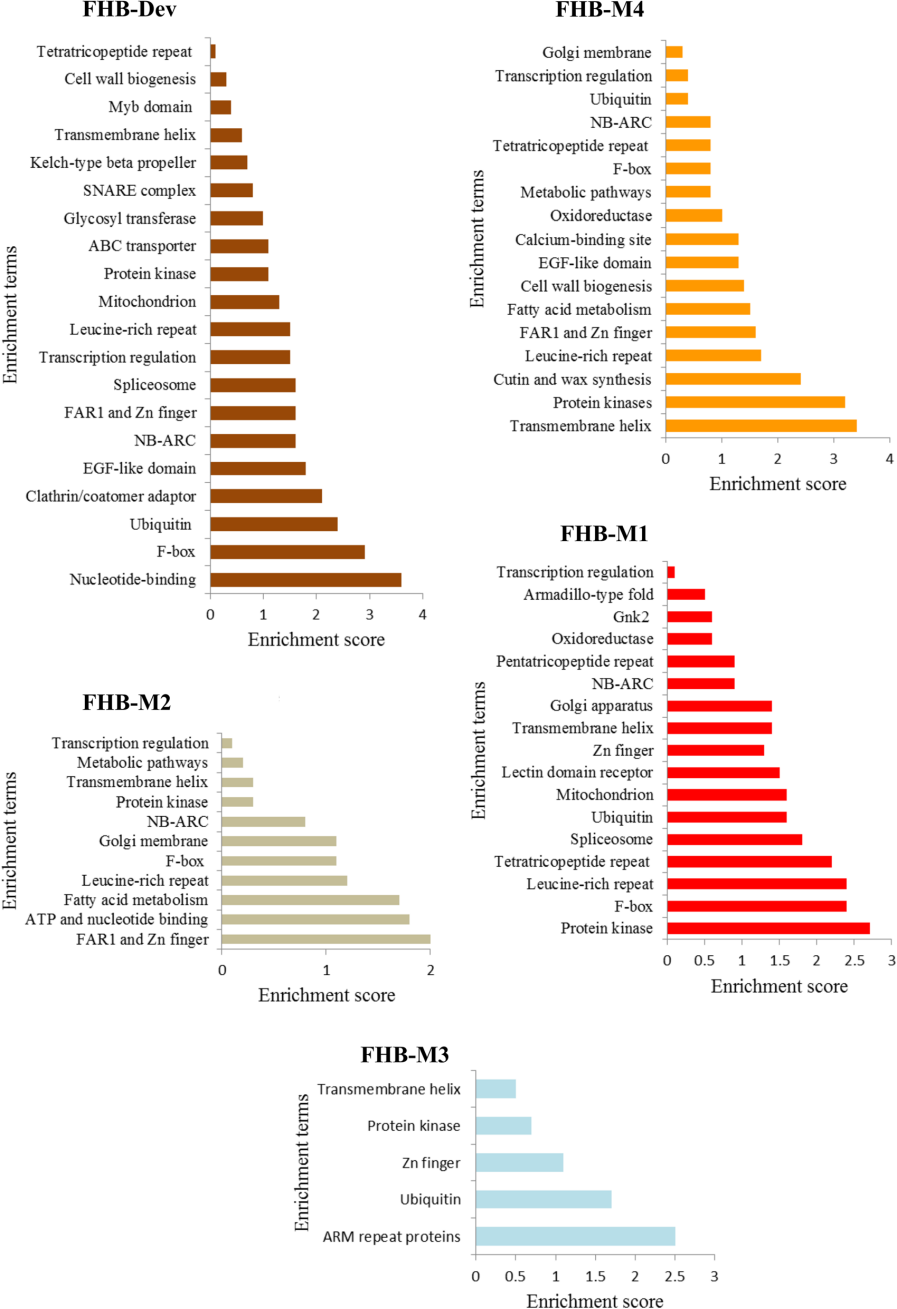
Functional annotation clustering of genes within modules significantly correlated with Type II FHB resistance. The modules significantly correlated with Type II FHB resistance were FHB-Dev, FHB-M4, FHB-M2, FHB-M1 and FHB-M3. Clustering of functional annotation was conducted with Database for Annotation, Visualization and Integrated Discovery (DAVID). All the presented clusters had Benjamini adjusted *P* < 0.05 when the *Arabidopsis thaliana* genome was used as background for enrichment analysis

Functional annotation clustering using DAVID software identified several significantly (Benjamini adjusted *P* < 0.05) enriched gene clusters for the modules significantly correlated with FHB resistance. Gene clusters identified in multiple modules had nucleotide binding (NB-ARC), leucine-rich repeat (LRR), F-Box, FAR1 and Zn finger, and protein kinase domains (Fig. 3). The NB-ARC and LRR are conserved domains present in plant resistance proteins which play a crucial role in effector triggered immunity (ETI) and effector triggered susceptibility (ETS) responses [71]. Genes with F-box domain are known for their function in protein-protein interaction and post-translational regulation through variable C-terminal domains such as the Kletch-type beta propeller (Kelch) repeat [72]. The role of F-box proteins in defense signaling has been repeatedly reported, e.g. by van den Burg et al. [73]. The FHB-Dev module was enriched in genes with Kelch repeat and F-box domains, likely due to the presence of modular genes carrying both F-Box and Kelch C-terminal domain. Far-Red Impaired Response 1 (FAR1) factors with Zn finger motifs have roles in flowering, light-regulated morphogenesis and response to biotic and abiotic stresses [74] that were over-presented in the FHB-Dev, FHB-M4 and FHB-M2 modules. Roles in both flowering and plant defense have been suggested for FAR1 genes, partially supporting a role for these genes in fine-tuning plant defense and development, which was supported here by the significant correlation of FHB-Dev module ME with plant height and maturity. Some protein kinases are involved in transducing signaling triggered by pathogen recognition and are required for activation of downstream defense responses [75]. The protein kinase gene cluster included several receptor-like kinases (RLKs). This class of kinases is known to serve as Pathogen-Associated Molecular Pattern receptors (PRRs) triggering Pattern Triggered Immunity (PTI) and in some instances as resistance genes for ETI [76].

An enriched gene cluster potentially linked with plant defense and unique to the FHB-Dev module contained genes with the clathrin/coatomer adaptor domain. Clathrins play a crucial role in regulating PTI and cell death by removing pattern-recognition receptor kinases/BRI1-associated kinase 1 (BAK1) co-receptors, such as EP receptor 1 (PEPR1), elongation factor Tu receptor (EFR), and Flagellin Sensing 2 (FLS2) from the surface through endocytosis [77]. The FHB-Dev module was also enriched in genes encoding ABC transporters. A role for ABC transporters in FHB resistance through enhancing tolerance to the mycotoxin DON has been suggested for *TaABCC3* [78] located on chromosome 3BS. There were at least four genes annotated as having ABC transporter activity in the FHB-Dev module located on chromosomes 2A, 4A and 4B (Additional file 3), which could be new candidate mycotoxin tolerance genes in wheat. A tentative enriched gene cluster with a role in defense and specific to the FHB-M4 module contained genes encoding cutin and wax synthesis proteins. A role for waxiness in FHB resistance was previously suggested and attributed to lower water availability for *F. graminearum* penetration on waxy spikelets [49]. Antifungal activity was proposed for *GnK2*, encoding plant-specific cysteine-rich proteins that appear in the FHB-M1 module as a significantly enriched gene cluster [79]. The only gene cluster specific to the FHB-M3 module contained genes with Armadillo (ARM) repeat domains which, similar to F-box proteins, are involved in protein-protein interactions and signaling associated with plant development and stress responses [80].

### Defense-related hub genes of modules correlated with FHB resistance

The genes involved at different layers of plant defense, including pathogen recognition, signaling pathways (kinases and phytohormones), and defense responses (antimicrobial proteins, secondary metabolites and regulators of reactive oxygen species (ROS) production and signaling) were considered as candidate defense genes per each of the five modules correlated with Type II FHB resistance (Additional file 3). Among those, genes with the top 10% intramodular connectivity or module membership (MM) were considered hub genes and described here; however, their function in FHB resistance must be confirmed using reverse genetic tools.

### FHB-M1 module

**Fig. 4:**
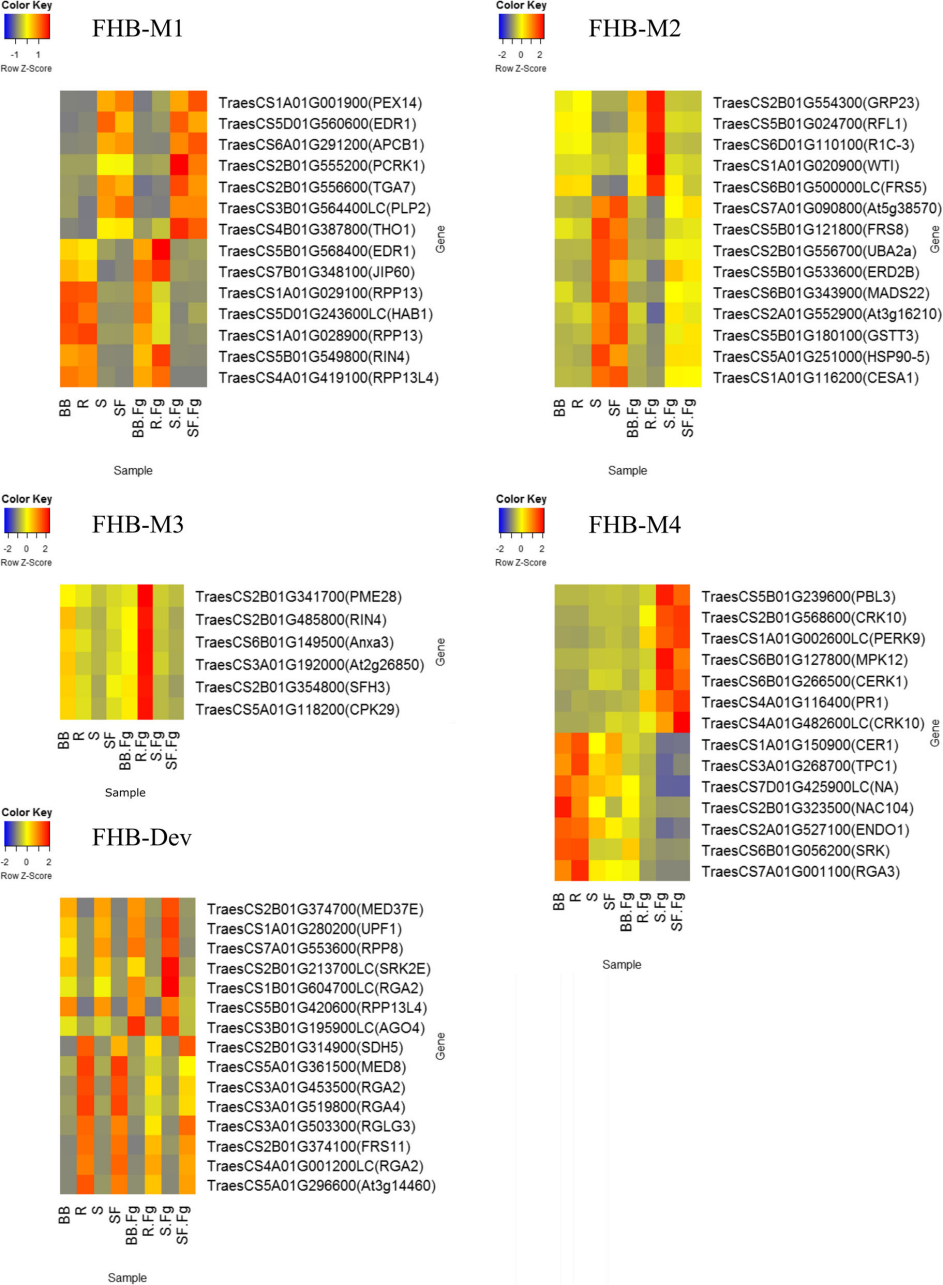
The candidate defense hub genes within modules significantly correlated with Type II FHB resistance. Genes with the top 10% intra-modular connectivity in modules significantly correlated with Type II FHB resistance (FHB-M1, FHB-M2 and FHB-M3, FHB-M4 and FHB-Dev modules) were considered as hub genes. Heat maps show the normalized counts value of each gene represented by a color spectrum ranging from red (high expression) to blue (low expression). The expression is shown for durum wheat cv. Strongfield (SF), *Triticum turgidum ssp. carthlicum* line Blackbird (BB) and two doubled haploid lines of the SF/BB population with transgressive resistance (R) and susceptible (S) FHB ratings, in mock-inoculated and *Fusarium graminearum* (Fg)-inoculated samples. Gene IDs were extracted from the International Wheat Genome Sequencing Consortium (IWGSC) RefSeq v1.0 annotation and gene names denoted in parenthesis belong to orthologues identified through blast search against the TrEMBL protein database

The FHB-M1 module hub genes potentially involved in the pathogen recognition encoded serine/threonine-protein kinase PCRK1 (PCRK1) and homologues of the disease resistance protein RPP13 (Table 1). The involvement of *PCRK1* as PRRs was proposed in Arabidopsis [81]. The expression of *PCRK1* was the highest in the inoculated S and SF spikes (Fig. 4), suggesting that *PCRK1* might be hijacked by the pathogen for induction of necrosis. Three orthologues of *RPP13* were detected, two located within the FHB resistance QTL on chromosome 1A and one on chromosome 4A within a locus that additively interacted with the FHB resistance QTL on chromosome 1A [19]. The expression of two genes encoding RPP13 (TraesCS1A01G029100 and TraesCS1A01G028900) was higher in R and BB than S and SF in both mock-inoculated and inoculated plants, consistent with their possible contribution to resistance. In contrast to other typical resistance proteins conferring resistance to biotrophs, RPP13 functions independently of Enhanced Disease Susceptibility 1 (EDS1) and non-race-specific disease resistance 1 (NDR1) proteins and does not require the accumulation of SA for defense signaling [82]. The uncharacterized pathway present downstream of RPP13 could be associated with the resistance of BB. The higher expression of transcription factor *TGA7* ortholog (TraesCS2B01G556600) that regulates the expression of genes downstream of SA signaling, in the S genotype suggests that the SA signaling pathway is likely linked with susceptibility. Previous studies suggested that some necrotrophs hijack resistance mechanisms effective against biotrophs to induce cell death, which promotes host cell colonization by necrotrophs [83, 84]. It is possible that BB uses orthologues of *RPP13* to sense pathogen invasion without triggering the SA signaling pathways and inducing cell death.

**Table 1:**
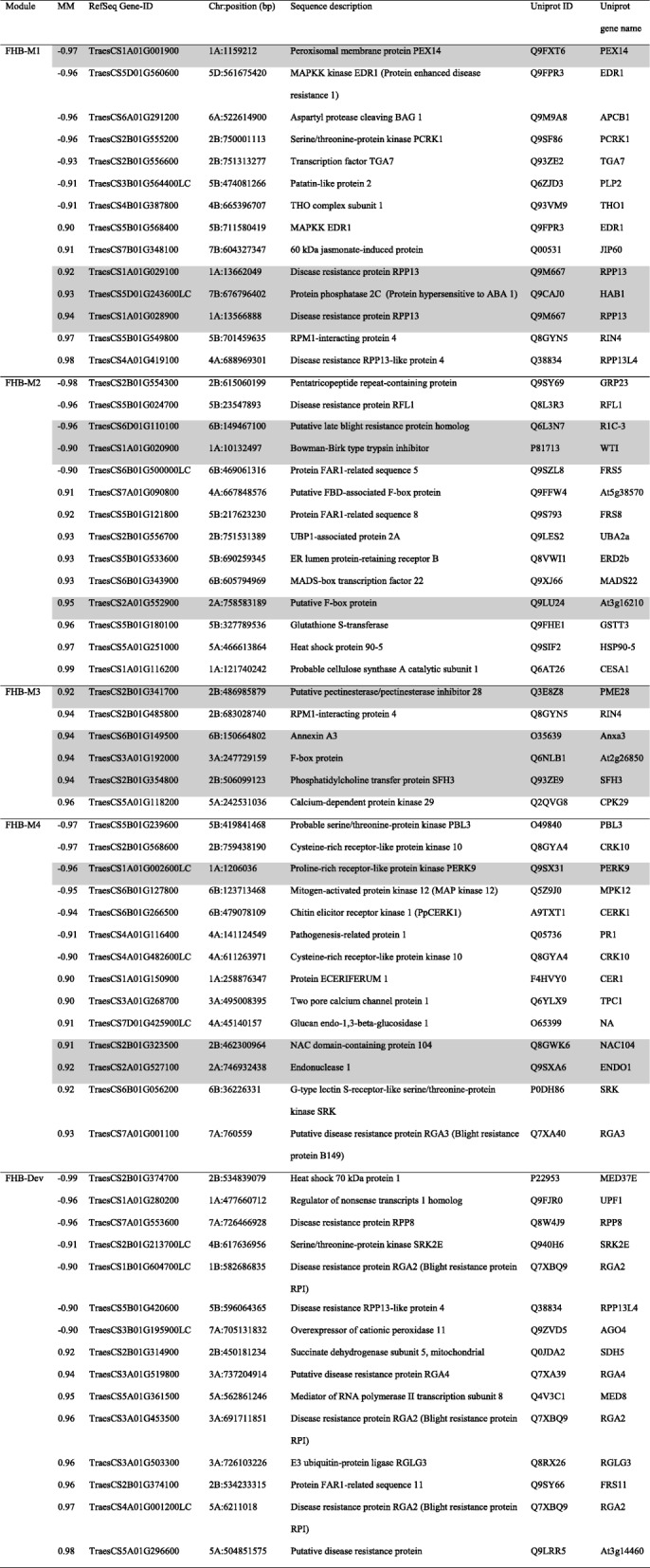
The chromosomal position and sequence description of candidate defense hub genes

Genes with the top 10% intramodular connectivity in gene networks significantly correlated with Type II FHB resistance (FHB-M1, FHB-M2, FHB-M3, FHB-M4 and FHB-Dev) are considered as hub genes. The module membership (MM) indicates the intramodular connectivity ranging between −1 to 1 and reflects correlation between the expression of module eigengenes (ME) and the module members (genes). The chromosomal position of genes (chr:position) was extracted from the International Wheat Genome Sequencing Consortium (IWGSC) RefSeq v1.0 assembly. Uniprot IDs and gene names were assigned to each gene from IWGSC RefSeq v1.0 annotation or by reciprocal blast search of the IWGSC RefSeq v.1.0 sequence against TrEMBL protein database. Highlighted genes were located within the interval of FHB resistance QTL reported for the cv. Strongfield/Blackbird population by Sari et al. [19]

An orthologue of *RPM1-interacting protein 4* (*RIN4*) was a hub gene in the FHB-M1 module. RIN4 is cleaved by a number of bacterial Type III effectors such as AvrRpm1 or AvrB as a mechanism for suppressing the PTI. RPM1 is a disease resistance protein that guards RIN4 and thus protects the plant against AvrRpm1-like effectors by inducing ETI responses [85]. This orthologue of *RIN4* in wheat (TraesCS5B01G549800) had higher expression in inoculated R and BB than S and SF genotypes. Ravensdale et al. [31] also reported the induction of a *RIN4* orthologue during the priming of FHB resistance in bread wheat using a *F. graminearum* isolate impaired in DON production. ETI has not been reported thus far in the *F. gramimearum*-wheat interaction. The presence of a functional RPM1/RIN4-like system in wheat, their association with RPP13 and resistance to FHB needs to be evaluated in future studies.

A FHB-M1 module hub gene located within the FHB resistance QTL on chromosome 1A encodes peroxisomal membrane protein PEX14 (PEX14). The orthologue of *PEX14* (TraesCS1A01G001900) had higher expression in SF and S than BB and R plants (Fig. 4). PEX14 is involved in peroxisome biogenesis [86]. The contribution of peroxisome to plant defense is through participation in biosynthesis of auxin, SA and JA [87]; however, a direct role in resistance to fungal pathogens has not yet been proposed for *PEX14*.

The presence of three FHB-M1 module hub genes within the FHB resistance QTL on chromosome 1A lends support to the association between this module and the QTL. The FHB-M1 module was not correlated with plant height and maturity (Fig. 1) and is likely associated with constitutive defense, as subtle differences between mock-inoculated and inoculated plants in the expression of the FHB-M1 module ME were observed (Fig. 2).

A FHB-M1 module hub gene was located within the FHB resistance QTL on chromosome 7B derived from SF. The orthologue of this gene encodes a hypersensitive to ABA 1 (HAB1) protein. *HAB1* has two splice variants playing contrasting roles in regulating the ABA signaling pathway in Arabidopsis [88]. The ABA signaling pathway triggers multifaceted defense responses in plants which vary with the type of plant tissues, the infection stage and the infection strategy of the pathogens [89]. Buhrow et al. [35] found a reciprocal cross-talk between the ABA and GA signaling that modulated FHB resistance. As the resistance allele of 7B QTL originated from SF [19] and the expression of *HAB1* orthologue (TraesCS5D01G243600LC) was lower in this than BB (Fig. 4), the lower expression of it might be linked to FHB resistance.

Orthologues of *MAPKK protein enhanced disease resistance 1* (*EDR1*; TraesCS5D01G560600 and TraesCS5B01G568400) were also hub genes in the FHB-M1 module. Surprisingly, short reads belonging to TraesCS5D01G560600 were mapped to the D genome of the IWGSC Refseq v1.0 assembly which is in theory absent in the tetraploid wheat genotypes used in this study. It is likely that these tetraploid genotypes have gained the D copy of *EDR1* through introgression occurred in lines derived from hybridization of hexaploid and tetraploid wheat. The *EDR1* copies on homologous chromosomes 5B (*EDR1-B*) and 5D (*EDR1-D*) had contrasting expression, with *EDR1-B* having higher expression in BB and R and *EDR1-D* expressed higher in the S and SF genotypes (Fig. 4). It is likely that BB and R carry different alleles of *EDR1-B* and *EDR1-D* from SF and S and that the homeologous alleles of *EDR1* act antagonistically for regulating defense, complying the epistasis interaction between homeologous genes. Previous studies suggested that EDR1 negatively regulates host cell death and suppresses the SA, ABA and ET signaling pathways [90–92]. EDR1 also functions in a MAP kinase cascade in concert with MPK3 and MPK6, allowing cross-talk between the SA, ABA and ET signaling. It is required for resistance to hemibiotrophic and necrotrophic fungal pathogens such as C*olletotrichum gloeosporioides*, *C. higginsianum* and *Alternaria brassicicola* through induction of defensins [93]. Genes encoding defensins were detected in the FHB-Dev module (TraesCS1A01G237500) with the highest expression in SF and in the FHB-M4 module (TraesCS1A01G050900) with the highest expression in BB (Additional file 3), supporting a cross-talk between FHB-M1, and FHB-Dev and FHB-M4 modules.

### FHB-M2 module

The FHB-M2 module hub genes tentatively involved in pathogen recognition encode orthologues of disease resistance protein RFL1 (RFL1) and late blight resistance protein R1C3 (R1C3) (Table 1). Both *RFL1* (TraesCS5B01G024700) and *R1C3* (TraesCS6D01G110100) had the highest expression in inoculated R plants (Fig. 4). *RFL1* is located within a cluster of resistance genes with *RPS5*, *RPS2* and *RPM1* in *A. thaliana* and shares sequence features with *RPS5* [94]. This may suggest that like RPS5, RFL1 guards protein kinase PBS1 that is targeted by numerous bacterial Type III effectors [95]. Orthologues of *PBS1* were detected in the FHB-Dev (TraesCS4B01G294300) and FHB-M4 (TraesCS5B01G239600) modules (Additional file 3), supporting the presence of a possible RFL1/PBS1-like interaction in the wheat-FHB pathosystem. *R1C3* confers resistance to isolates of *Phytophthora infestans* carrying *Avr1* [96]. The higher expression of this gene in R plants (Fig. 4) and its co-localization with the FHB resistance QTL on chromosome 6B (derived from BB; Table 1) is consistent with its involvement in resistance.

The orthologue of *Bowman-Birk type trypsin inhibitor* (*WTI*) is a hub gene of the FHB-M2 module (Table 1). *WTI* encodes a serine protease with demonstrated antimicrobial activity [97]. The higher expression of *WTI* orthologue (TraesCS1A01G020900) in R plants (Fig. 4) and its co-localization within the FHB resistance QTL on chromosome 1A support a role in resistance. A putative F-box protein that is an orthologue of *At3g16210* in *A. thaliana* co-located with the FHB resistance QTL on chromosome 2A (Table 1). Inoculated R plants had lower expression of the *At3g16210* orthologue (TraesCS2A01G552900) than the other genotypes (Fig. 4) which suggests the lower expression is associated with the transgressive resistance of R plants.

The FHB-M2 module hub transcription factors were orthologues of UBP1-associated protein 2A (*UBA2a*), MADS-box transcription factor 22 (*MADS22*), and protein FAR1-related sequence 5 and 8 (*FRS5* and *FRS8*) (Table 1). *UBA2a* regulates the turnover of mRNAs in the nucleus and is localized in nuclear bodies in response to ABA signaling [98]. The expression of the *UBA2a* orthologue (TraesCS2B01G556700) was higher in S and SF than other genotypes in the mock-inoculated plants, but the difference between genotypes was negligible in inoculated plants (Fig. 4). This suggests that infection represses the *UBA2a* expression in S and SF. The detection of *UBA2a* and *HAB1* as hub genes corroborates the involvement of ABA signaling in the reaction of the tetraploid wheat genotypes to FHB. MADS-box transcription factors regulate developmental traits such as flowering time as well as stress-related responses such as abscission and senescence [99]. Khong et al. [99] identified a MADS-box protein acting as hub gene upstream of several stress related pathways that negatively regulated resistance to the rice pathogens *Magnaporthe oryzae* and *Xanthomonas oryzae*. The higher levels of resistance in BB and R compared to other genotypes could also be linked to the lower expression of MADS22 orthologue (TraesCS6B01G343900). The ortologues of *FRS5* (TraesCS6B01G500000LC) and *FRS8* (TraesCS5B01G121800) had contrasting expression pattern, with the orthologue of *FRS5* having the highest expression in R and *FRS8* in SF (Fig. 4). A negative regulation of defense through integrating chlorophyl biosythesis and SA signaling was proposed for FAR1 genes as the null mutants of Arabidopsis had higher levels of ROS and SA and were more resistant to *Pseudomonas syringae* [100]. Positional cloning of the wheat vernalization gene *VRN1* identified a MADS-box genes (*AP1*) which interacts epistatically with *VRN2* gene for regulating vernalization and flowering time traits in wheat [101]. While studying the involvment of *VRN-B1* in control of heading date, Kiseleva et al. [102] identified an orthologue of FAR1 as a candidate heading data gene. An orthologue of *FRS11* was present in the FHB-Dev module that was highly correlated with plant maturity traits. This gene was located within the interval of the FHB resistance QTL on chromosome 2B derived from SF (Table 1), supporting a possible association of *FAR* genes with resistance. The pleiotropic effects of *MADS22*, *FRS5, FRS8* and *FRS11* on developmental and FHB resistance traits could be a valid cause for the association between these traits in multiple previous studies [19, 103]. The co-localization of FHB resistance QTL with plant maturity is often interpreted as the contrubution of late maturity traits to disease escape. By contrast, the results of this study suggests an intricate physiological involvment of maturity genes in the wheat-FHB interaction which requires future further validation.

An orthologue of *endoplasmic reticulum lumen protein-retaining receptor B* (*ERD2b)* is a FHB-M2 module hub gene (Table 1). *ERD2b* expression is required for the biogenesis of EFR receptor involved in recognition of the bacterial PAMPs, elf19 and flg22 [104] and for the induction of programmed cell death through retrograde pathway from the Golgi to the endoplasmic reticulum [105]. Silencing *ERD2b* delayed cell death induced by *Xanthomonas oryzae* pv. *oryzae* and *Pseudomonas syringae* pv. *tomato* DC3000 [105], suggesting a role in cell death regulation. The expression of a *ERD2b* orthologue (TraesCS5B01G533600) was the highest in mock-inoculated S and lowest in inoculated R plants (Fig. 4), suggesting its negative effect on FHB resistance probably through interfering with the biogenesis of the PPRs, promoting the induction of cell death and susceptibility to FHB. The higher expression of the *ERD2b* orthologue could be also a response to widespread ETS in the S line requiring the deployment *ERD2b* and its ligands involved in the ER quality control to alleviate resulting ER stress.

An orthologue of *CESA1* (TraesCS1A01G116200) is the only FHB-M2 module hub gene with a role in cell wall modification (Table 1). In addition to its role in cell wall modification, a role in pathogen recognition has recently been proposed for *CESAs* [106]. Ramírez et al. [106] suggested a role in surveillance of cell wall integrity for these genes allowing plants to sense *Botrytis cinerea* invasion and to transduce defense signaling pathways. These authors proposed the association of lower expression with resistance since necrotrophs require cellulose to generate glucose as a food source. The expression of the *CESA1* orthologue was lower in BB and R than SF and S inoculated plants (Fig. 4), supporting the association of lower *CESA1* expression with resistance.

### FHB-Dev module

The expression pattern of FHB-Dev module ME suggested that it is likely associated with the partial resistance of SF (discussed above). The co-localization of three FHB-Dev module hub genes with the FHB resistance QTL on chromosome 2B derived from SF (Table 1) further supports this association. The orthologue of these hub genes encoded heat shock 70 kDa protein 1 (MED37E), succinate dehydrogenase subunit 5 (SDH5) and FRS11. A role for *MED37E* in resistance to the downy mildew pathogen *Hyaloperonospora parasitica* has been proposed [107]. The expression of *MED37E* orthologue (TraesCS2B01G374700) was the highest in the inoculated S plants (Fig. 4), suggesting the involvement of *MED37E* in susceptibility. *SDH5* is involved in ROS generation in mitochondria and has multiple roles in plant development and stress response [108]. The orthologue of *SDH5* (TraesCS2B01G314900) had higher expression in the inoculated SF than in the other genotypes. This and the co-localization of the gene with the FHB resistance QTL on chromosome 2B suggests the involvement of ROS production and signaling in reaction of SF to *F. graminearum* infection.

There were seven orthologues of resistance genes encoding disease resistance protein RPP8 (RPP8), blight resistance protein RPI (RGA2, three genes), disease resistance RPP13-like protein 4 (RPP13-L4), putative disease resistance protein RGA4 (RGA4) and putative disease resistance protein At3g14460 (At3g14460) in the FHB-Dev module (Table 1). The orthologues of *RPP8*, *RGA2* (TraesCS1B01G604700LC), and *RPP13L4* (TraesCS5B01G420600) had the highest expression in the inoculated S plants while the other four genes had the highest expression in inoculated SF plants (Fig. 4). Except for TraesCS1B01G604700LC, the other orthologues of *RGA2* had higher expression in SF. RGA2 and 4 are members of a four gene cluster in *Solanum bulbocastanum* mediating broad spectrum resistance against *Phytophthora infestans* [109]. Their presence within the same gene cluster in wheat is unlikely since the orthologues were located on different chromosomes of wheat. The concerted action of these genes in wheat-FHB interaction is not clear and needs to be investigated.

A FHB-Dev module hub gene encoded serine/threonine-protein kinase SRK2E (SRK2E) (Table 1). SRK2E functions in the ABA signaling pathway induced downstream of bacterial PAMP recognition and is required for ABA-mediated stomatal closure [49]. *SRK2E* regulates the ABA signaling pathway in concert with *HAB1*. The expression of the *SRK2E* orthologue (TraesCS2B01G213700LC) was the highest in inoculated S, in contrast to *HAB1*, which had the highest expression in inoculated R and BB genotypes (Fig. 4). This is consistent with the contrasting roles of *HAB1* and *SRK2E* in ABA signaling, where *HAB1* positively and *SRK2E* negatively regulates the pathway [49, 110]*.* It is likely that ABA signaling is associated with susceptibility and that the negative regulation of ABA by *HAB1* is linked to resistance.

An orthologue of E3 ubiquitin-protein ligase RGLG3 (*RGLG3*) was a hub gene in the FHB-Dev module (Table 1). *RGLG3* mediates upstream regulation of JA signaling and suppresses the SA signaling pathway [111, 112]. Zhang et al. [112] proposed the hijacking of *RGLG3* by the *F. verticillioides* mycotoxin fumonisin B1 for induction of cell death. The higher expression of the *RGLG3* orthologue (TraesCS3A01G503300) in SF (Fig. 4) might be linked with activation of the JA signaling pathway and the delayed cell death, hence providing some levels of tolerance to FHB in this genotype.

As expected, several gene associated with regulation of developmental traits were among hub genes of FHB-Dev module (Additional file 3), supporting the correlation of the ME with plant height and relative maturity (Fig. 1). For example, an orthologue of *transcriptional co-repressor SEUSS* (*SEU*), a hub gene with MM = 0.95, had higher expression in R and SF than the other genotype. SEU is a transcription repressor and is induced in response to auxin signaling [113]. SEU forms a physical complex with the LEUNIG transcriptional coregulator to repress Arabidopsis transcription required for switching to flowering phase [113]. Two orthologues of *casein kinase 1-like protein HD16* (*HD16*) were hub genes of FHB-Dev module (MM = 0.97 and 0.96; Additional file 3). HD16 is involved in post-translational regulation of flowering time through GA signaling, and had higher expression in R and SF than the other genotypes (Additional file 3). The presence of both defense and developmental hub genes in the FHB-Dev module confirm an interwoven association between FHB resistance and developmental traits in wheat [34].

### FHB-M3 module

All the hub genes in the FHB-M3 module had their peak expression in the inoculated R plants (Fig. 4), corroborating their potential contribution to the transgressive expression of resistance. Four FHB-M3 hub genes located within the interval of reported FHB resistance QTL in the SF/BB population (Table 1). An orthologue of pectinesterase/pectinesterase inhibitor 28 (*PME28*) was within the interval of the FHB resistance QTL on chromosome 2B derived from SF. Marzin et al. [114] found no evidence for the direct involvement *PME28* in resistance of barley to *Rhynchosporium commune*. However, a pectinesterase inhibitor gene mediated resistance of cotton to *Verticillium dahliae* through disrupting the activity of fungal polygalactronase [115]. A FHB-M3 hub gene co-located with the FHB resistance QTL on chromosome 6B is an orthologue of *Annexin A3* (*Anxa3*). Accumulation of annexins in plants is associated with tolerance to various biotic and abiotic stresses [116]. A FHB-M3 module hub gene encoding for an F-box protein co-located with the FHB resistance QTL on chromosome 3A. The potential role of F-box proteins in defense signaling and post-translational regulation of defense was discussed above. An orthologue of phosphatidylcholine transfer protein *SFH3* was among the FHB-M3 module hub genes co-located with the FHB resistance QTL on chromosome 2B. *SFH3* encodes a lipid transfer protein (LTP) to which several roles in plant immunity have been assigned, e.g. early recognition of pathogen attacks [45, 117].

### FHB-M4 module

FHB-M4 module hub genes encoded pathogen recognition receptors such as chitin elicitor receptor kinase 1 (CERK1) (Table 1). CERK1 is a lysine motif (LysM) receptor-like kinase involved in recognition of carbohydrate ligands and triggers PTI responses [118]. Previous research indicated that PAMP recognition mediated through CERK1 triggers MAPK cascades through PBS1 like (PBL) receptor kinases that also guards the resistance genes, *RFL1* and *R1C3,* which were the hub genes of the FHB-M2 module. Interestingly, orthologues of *PBL3* (*PBL3*) and *mitogen-activated protein kinase 12* (*MAPK12*) were hub genes of the FHB-M4 module, supporting the notion that *CERK1* and *PBL3* are involved in the activation of PTI responses in the genotypes used in this study. This is further supported by the very similar expression patterns of *CERK1*, *PBL3* and *MAPK12* orthologues (TraesCS6B01G266500, TraesCS5B01G239600 and TraesCS6B01G127800), with the highest expression levels recorded in inoculated SF and S (Fig. 4). The pathogen might use *CERK1*/*PBL3* to promote cell death in SF and S as suggested by Petutschnig et al. [119], and lower expressions of these might be linked to higher levels of FHB resistance in BB and R. A previous study implicated *CERK1* in the induction of pathogenesis related 1 (PR1) and the SA signaling pathway which is supported here by the co-expression of *PR-1* with *CERK1* and *PBL3* in FHB-M4 module. This is consistent with the possibility of hijacking of the cell death pathway by *F. graminearum* through triggering *CERK1*-mediated SA signaling.

A FHB-M4 module hub gene that co-located with the FHB resistance QTL on chromosome 1A encodes an orthologue of proline-rich receptor-like protein kinase PERK9 (Table 1) that regulates root growth in Arabidopsis [120]. The similarly higher expression of *PERK9* orthologue (TraesCS1A01G002600LC) in inoculated S and SF than BB and R plants (Fig. 4) suggests that it might be involved in susceptibility to FHB. The role of *PERK9* in perceiving PAMPs or pathogen effectors remains to be elucidated. An orthologue of NAC domain-containing protein 104 (*NAC104)* was among the FHB-M4 module hub genes co-located with the FHB resistance QTL on chromosome 2B. NAC104 is a transcription factor that negatively regulates cell death during vascular development [121]. Mclellan et al. [122] reported that a *P. infestans* effector prevents the re-localization of two NAC transcription factors from the endoplasmic reticulum to the nucleus as a virulence mechanism. The expression of *NAC104* orthologue (TraesCS2B01G323500) was higher in inoculated BB than the other genotypes (Fig. 4), supporting a role in resistance.

### Assessing the expression of candidate defense hub genes using qRT-PCR

**Fig. 5:**
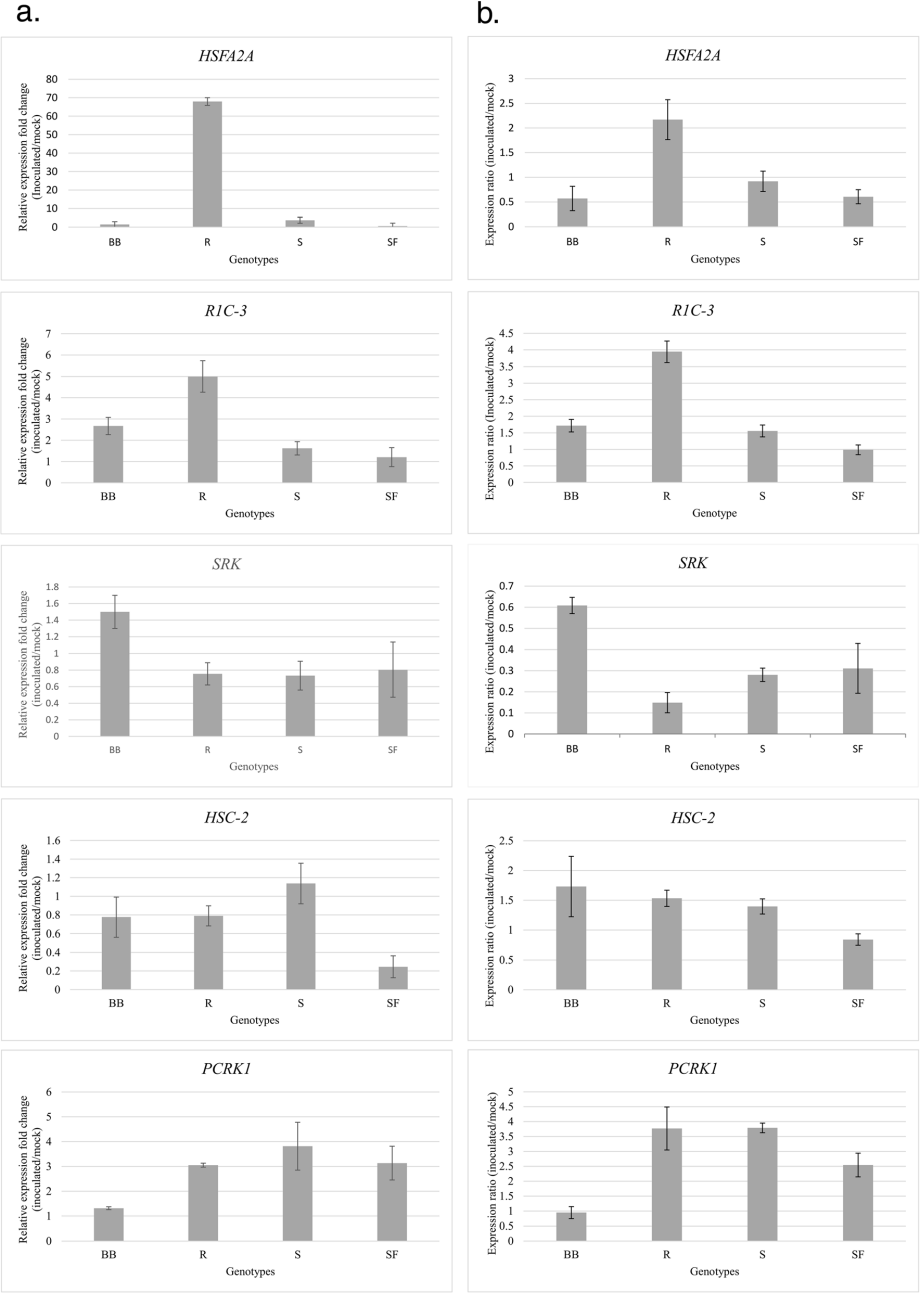
The expression fold change of selected candidate defense hub genes determined by quantitative real time PCR (**a**) and by RNA sequencing (**b**). For qRT-PCR, the expression level of *Triticum turgidum ssp. carthlicum* Blackbird (BB), durum wheat cv. Strongfield (SF), and doubled haploid lines from the SF/BB population with transgressive resistant (R) and susceptible (S) inoculated with *Fusarium graminearum* was reported as expression fold change relative to mock inoculated samples. QRT-PCR data were normalized using α-*tubulin* gene expression as a reference gene. The expression ratio of same samples from RNA-sequencing was calculated by dividing the normalized read counts of the inoculated to the average read counts of mock-inoculated samples. Errors bars show the standard deviation of the means. The candidate hub genes encode heat stress transcription factor A-2a (HSFA2A), putative late blight resistance R1C-3 (R1C-3), G-type lectin S-receptor-like serine/threonine-protein kinase SRK (SRK), heat shock cognate 70 kDa protein 2 (HSC-2) and serine/threonine-protein kinase PCRK1 (PCRK1)

The overall correlation between the relative expression fold changes obtained using qRT-PCR and the expression ratio obtained from RNA sequencing was 70% (*P* = 0.0008). Similar to the results of RNA-seq analysis (Fig. 5b), genotypes differed in the expression levels reported using qRT-PCR of all the five selected candidate defense hub genes (Fig. 5a). The results of qRT-PCR confirmed that orthologues of *heat stress transcription factor A-2a* (*HSFA2A)* and *R1C-3* had higher expression in R while *G-type lectin S-receptor-like serine/threonine-protein kinase SRK* (*SRK)* was expressed at higher levels in BB than the other genotypes. *Heat shock cognate 70 kDa protein 2* (*HSC2*) had lower expression in SF and *PCRK1* in BB than the other genotypes.

We initially tested three reference genes as proposed by Paolacci et al. [64], in order to use the geometric average of multiple reference genes for normalization. Only TraesCS4A02G065700 met the required amplification efficiency for our assays. A higher amount of correlation between the two techniques might have been achieved if multiple reference genes were used. Nevertheless, the high correlation between the results from the two techniques supports the analytical and technical accuracy of RNA-seq. A similar level of correlation was previously reported by De Cremer [123] when analyzing the lettuce and *B. cinerea* interaction using RNA-seq and qRT-PCR.

### Genetic variants within the candidate defense hub genes

The identified genetic variants within the candidate hub genes are presented in Additional file 4. High-impact polymorphisms were found within four candidate hub genes (Table 2). *PEX14* had two SNPs at splice acceptor sequences, suggesting that BB and SF had splicing variations in this gene. A high-impact variant within orthologue of *RGA4* (TraesCS3A01G519800) imposed pre-mature stop codon, providing that BB has a truncated version of RGA4. The orthologue of *At3g14460* (TraesCS5A01G296600) had a high-impact frame-shift variant. Similar to *RGA4*, *At3g14460* encodes a resistance protein, further supporting the role of resistance proteins in the wheat-*F. graminearum* interaction, however their contribution to resistance/susceptibility to FHB must be examined in the future. Orthologue of *CESA1* (TraesCS1A01G116200) also carried a high-impact frame-shift variant. As discussed above, *CESA1* plays a role in resistance to necrotrophs by surveying the cell wall integrity, sensing the pathogen invasion and transducing defense signals. The presence of a high-impact genetic variant in *CESA1* supports its role in FHB resistance.

**Table 2:**
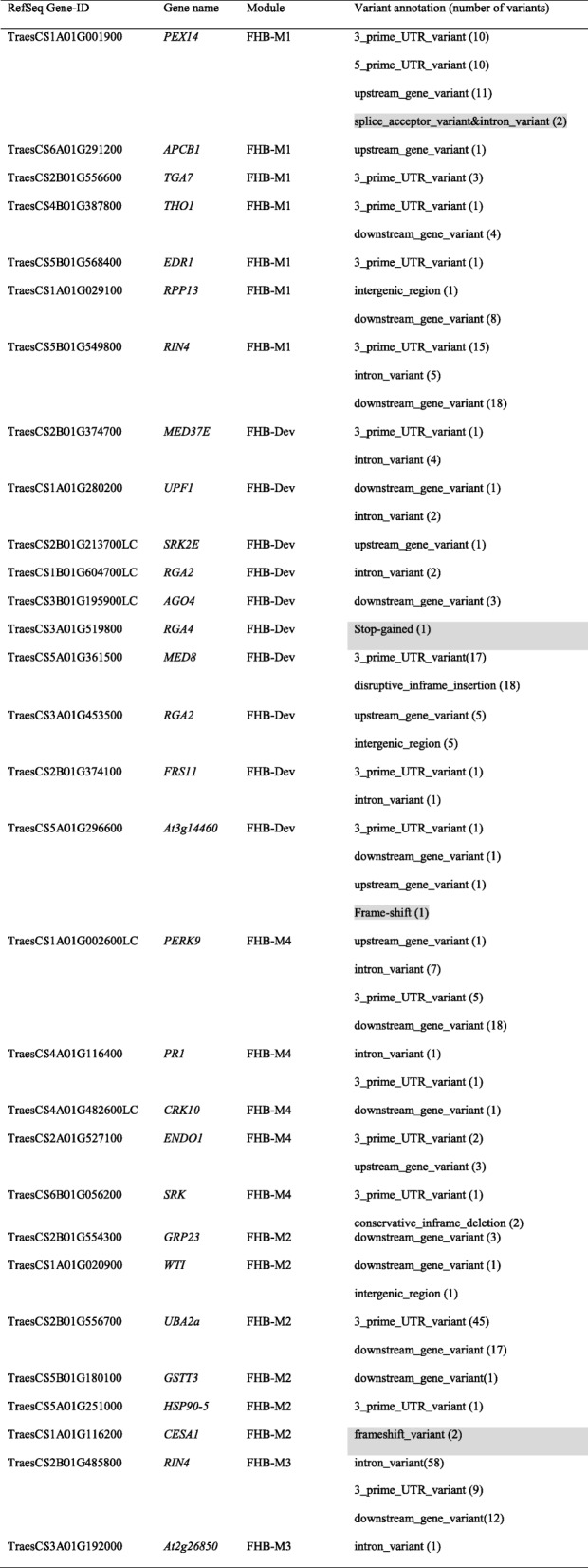
The genetic variants identified within the candidate defense hub genes of modules significantly correlated with Type II FHB resistance

Genes with the top 10% intramodular connectivity in gene networks significantly correlated with Type II FHB resistance (FHB-M1, FHB-M2, FHB-M3, FHB-M4 and FHB-Dev) are considered hub genes. Only variants annotated with modifier and high-impact ontology terms are shown and high-impact variants are highlighted in grey

Genotypes showed a high number of genetic variants in *RIN4* orthologues (TraesCS5B01G549800 and TraesCS2B01G485800). This, along with their differential expression among genotypes, stresses the potential involvement of *RIN4* in the wheat-*F. graminearum* interaction. *RIN4* is at the forefront of interaction with several pathogenic effectors [85], exerting a high amount of selective pressure on this gene. The high amount of sequence variation in this gene between BB and SF could affect recognition of RIN4 by *F. graminearum* effectors in BB as a mechanism to escape ETS response induced downstream of RIN4. Genotypes showed high amounts of sequence variation in *PERK9* and *UBA2a*. The genetic variants in *PERK9* were mostly located in the downstream genic region. Receptor-like kinases often carry an intracellular kinase domain in the downstream genic (C terminal) region [124]. Blackbird and SF are thus variable at the C terminal domains of *PERK9* which theoretically modify *PERK9* function in transducing signal after PAMP recognition by its transmembrane N terminal domain. *UBA2a* carried 45 genetic variants in the 3′ untranslated region (3′UTR). The 3′UTR often contains post transcription regulator elements. The role of *UBA2a* in regulating ABA signaling and the presence of a high number of SNPs between BB and SF in the 3′UTR support a potential role for ABA signaling in the wheat-*F. graminearum* interaction. *MED8* carried 17 genetic variants in the 3′UTR and 18 disruptive in-frame insertion variants. *MED8* encodes a mediator protein complex (adaptor between transcription factor and RNA-polymerase II) required for JA signaling, resistance to necrotrophs such as *F. oxysporum*, and flowering in Arabidopsis [125]. The study by Kidd et al. [125] also supported the similar function of the *MED8* homologue in wheat, lending further support for the involvement of this gene in JA signaling in wheat. The notion that this gene also confers flowering date in Arabidopsis is consistent with the association of flowering genes such as *FRSs* and *MED8* and the modification of resistance to necrotrophic pathogens.

### General discussions and conclusion

Candidate hub genes with receptor activity belonged mostly to the NBS-LRR gene family. To our knowledge, ETI has not been implicated in the wheat-*F. graminearum* interaction, corroborating that the NBS-LRR genes might be targeted by *F. graminearum* pathogenic effectors for the induction of cell death. This explains the higher expression of the NBS-LRR genes of the FHB-Dev modules, including orthologues of *RPP8*, *RGA2* (three paralogues), *RGA4* and *At3g14460* in the susceptible rather than resistant genotypes. Higher expression of the *CERK1*/*PBL3* co-receptor in the S line supports that *F. graminearum* pathogenic effectors might also hijack PAMP receptors and hence resistance in BB and R is linked to lower *CERK1*/*PBL3* expression. The observation that orthologues of *RIN4* and *PBS1* were detected as hub genes in this study supports the existence of an indirect interaction between *F. graminearum* effectors and the NBS-LRR genes following the decoy/guard gene-for-gene interaction model [126], leading likely to ETS. The orthologues of *RIN4* detected on chromosome 5B and 2B carried large amount of sequence variation between BB and SF. It is likely that the presence of large number SNPs in *RIN4* affects its affinity for some of the *F. graminearum* effectors, rendering BB less sensitive to the *F. graminearum* virulence factors. Clustering of samples used for gene co-expression analysis based on the expression of the whole transcriptome suggested that BB differed from other genotypes, having fewer transcriptional changes post-infection. This could be attributed to the sequence variation in candidate receptor genes such as *RIN4* that allows BB to be less sensitive to the *F. graminearum* virulence factors. Cell death inhibition could be achieved through the activity of genes encoding clathrins acting as negative cell death feedback loop by removing pattern-recognition receptor kinases/BAK1 co-receptors from the cell surface. In addition, *ERD2b* is involved in biogenesis of ERF receptor and had the lowest expression in R plants meaning lower availability of the PRRs in the plasma membrane of BB. These altogether suggest that the resistance genotypes might be equipped with a mechanism to remove PRRs from the cell surface to escape the recognition of *F. graminearum* pathogenic effectors. However, this cannot explain the higher expression of *RPP13* and *RFL1* in the resistant genotypes. According to previous studies, some necrotrophs hijack the SA signaling pathway for inducing cell death [84]. The association of *RPP13* and *RFL1* expression with resistance could be linked to their difference from typical resistance proteins by the ability to transduce an unknown SA-independent signaling pathway, allowing resistant genotypes to express resistance without inducing SA signaling. The function of NBS-LRR and PRRs in resistance to FHB remains a relevant topic for future studies.

Several known regulatory genes of the ABA signaling pathway including *HAB1*, *UBA2a*, and *SRK2E*, were identified as candidate hub genes in this study, supporting the involvement of ABA signaling in regulating defense responses to FHB. The presence of a high amount of sequence variation between resistant and susceptible genotypes at *UBA2a* and its higher expression in susceptible genotypes supports a role for *UBA2a* in susceptibility. *HAB1* had the highest expression in the R genotype and *SRK2E* in the S genotype, indicating that negative regulation of the ABA signaling by *HAB1* might be associated with resistance. Considering that *HAB1* and *SRK2E* work antagonistically for regulating the ABA signaling, their contrasting expression levels in R and S plants support further the regulatory role of *HAB1*/*SRK2E* in this pathosystem. The detection of two homeologous copies of *EDR1* as hub genes supports a role for these genes in the wheat-*F. graminearum* interaction. *EDR1* is a hub gene involved in the MAP kinase cascade and mediates cross-talk between the ABA, SA and JA signaling pathways in Arabidopsis [93]. The contrasting expression of the homeologous copies of *EDR1* could be explained by their roles in regulating resistance in the genotypes studied. *EDR1* might confer FHB resistance through regulating cell death and inducing the expression of antifungal peptides such as defensins. *PEX14* had higher expression in the susceptible genotypes, corroborating higher engagement of the peroxisome in the susceptible than resistant genotypes. The peroxisome plays a crucial role in the biosynthesis of several plant hormones, especially JA and auxin, and the detoxification of ROS [126]. The presence of high-impact genetic variants in *PEX14* supports a function for this gene in the pathosystem studied here.

Transcription factors detected as hub genes were orthologues of *MADS22*, *FRS5*, *8* and *11*. These genes are known to pleiotropically modulate plant defense and developmental traits. For example, member of MADS and FRS transcription factors include the known *VRN2* candidate genes [101, 102]. Previous mapping studies using the SF/BB population identified FHB resistance QTL co-located with plant height and relative maturity [19], suggesting that FHB resistance is associated with these traits in BB and SF. Significant FHB-Dev module correlation with Type II FHB resistance, plant height, and maturity supports this association. Understanding the network of regulatory genes modifying FHB resistance and developmental traits is required for devising novel methods for breeding highly resistant durum varieties.

Several genes known to negatively regulate cell death, including *NAC104*, *ENDO1*, *EDR* and *Anxa3*, had higher expression in the resistant genotypes. Samples used for WGCNA analysis were collected at 48 h post infection, which is often the time that *F. graminearum* ends its biotrophic phase by secreting necrosis-inducing effectors. The coincidence of this with the higher expression of genes involved in cell death inhibition in the more resistant genotypes is consistent with the contribution of these genes to resistance. Breeding durum lines capable of inhibiting the switch to the necrotrophic phase is challenging; however, this seems to be an important strategy for developing desirable levels of resistance. Using non-hazardous chemicals to prime or induce anti-apoptotic genes seems a promising strategy for reducing the damage triggered by FHB disease, and needs to be evaluated for the control of FHB.

Previous studies suggested an association between cell wall composition and FHB resistance in durum wheat [50]. The orthologue of *PME28*, a candidate gene co-located with the FHB resistance QTL on chromosome 2B, encodes a pectinesterase inhibitor that reinforces the plant cell wall against fungal polygalactronase activity. Its higher expression in the more resistant genotypes lends support to its involvement in resistance. An orthologue of *CESA1* had lower expression in resistant plants. Lower *CESA1* expression might lead to lower cellulose deposition in the cell wall, which probably reduced sugar availability to the fungus during the early phase of infection and retarded its growth. *CESA1* also plays a role in monitoring cell wall integrity and signaling, making it a candidate FHB resistance gene. Preformed and induced physical barriers are important components of quantitative FHB resistance. Breeding for these traits is desired since a broad spectrum resistance against multiple pathogens might be achieved.

## Conclusions

The difference between the resistant and susceptible genotypes in deploying defense related transcripts at several layers of plant defense machinery, including recognition, signaling and defense pathway regulation was highlighted in this study. Gene network analysis allowed identification of candidate regulator genes and genes associated with constitutive resistance, those that might be difficult to detect using traditional differential expression analysis. This study also shed light on the association of developmental traits with FHB resistance and partially explained the co-localization of FHB resistance with plant height and maturity QTL reported in several previous studies. It also identified candidate genes within the FHB resistance QTL reported by Sari et al. [19] on chromosomes 1A (*PEX14*, *RPP13* [2 orthologues], *WTI*, *PERK9*), 2B (*MED37E*, *SDH5*, *FRS11*, *PME28*, *SFH3*, *NAC104* and *ENDO1*) and 6B (*R1C*-3 and *Anxa3*). It delivered SNPs within most of these candidate genes for future mapping studies. Moving forward, the SNPs within the candidate hub genes will be used for high-resolution mapping of FHB resistance QTL in BB and SF using NILs carrying recombination break points in the FHB resistance QTL interval. SNPs within the candidate genes will also be validated for utilization in breeding programs.

## Supplementary information

**Additional file 1. **Hierarchical clustering of gene expression in samples used for weighted gene co-expression network analysis. Genotypes are durum wheat cv. Strongfield (SF), *Triticum turgidum ssp. carthlicum* line Blackbird (BB) and a resistant (R) and a susceptible (S) doubled haploid line of the SF/BB population. Samples that were inoculated with *Fusarium graminearum* have “.Fg” suffix. Samples are numbered sequentially to represent the three biological replicates per treatment. Marked in red is an outlier sample excluded from weighted gene co-expression network analysis.

**Additional file 2. **Primer pairs used for quantitative real time PCR of selected candidate hub genes. The candidate hub genes selected encode heat stress transcription factor A-2a (HSFA2A), putative late blight resistance R1C-3 (R1C-3), G-type lectin S-receptor-like serine/threonine-protein kinase SRK (SRK), heat shock cognate 70 kDa protein 2 (HSC-2) and serine/threonine-protein kinase PCRK1 (PCRK1). Expression data were normalized using α*-tubulin* as reference gene. Gene-IDs are from International Wheat Genome Sequencing Consortium (IWGSC) RefSeq v1.0 annotations. Genes belonging to various gene co-expression networks (modules) were tested.

**Additional file 3.** Candidate defense genes of the five gene co-expression networks (modules) significantly correlated with Type II FHB resistance.

**Additional file 4.** Genetic variants identified within and in 5′ and 3′ un-translated region around candidate hub genes identified in five gene co-expression networks (modules) significantly correlated with Type II FHB resistance.

## Data Availability

The paired-end Illumina RNA-sequencing reads are deposited in the Sequence Read Archive (SRA) of the National Center for Biotechnology Information (NCBI) under BioProject accession PRJNA531693 (https://www.ncbi.nlm.nih.gov/Traces/study/?acc=PRJNA531693). Biosamples are named with “BB” for Blackbird, “SF” for Strongfield, “E872” for the transgressive resistant and “C679” for transgressive susceptible double haploid lines of the SF/BB population. All the other data generated and analyzed during this study are included in this article or its supplementary files.

## Abbreviations

ABA: Abscisic acid
BB: *Triticum turgidum ssp. carthlicum* line Blackbird
DH: Doubled haploid
ETH: Ethylene
ETI: Effector-triggered immunity
ETS: Effector-triggered susceptibility
Fg: *Fusarium graminearum*
FHB: Fusarium head blight
GA: Gibberellic acid
IWGSC Ref Seq: International Wheat Genome Sequencing Consortium Reference Genome Sequence
JA: Jasmonic acid
MAS: Marker-assisted selection
ME: Module eigengene
MM: Module Membership
PTI: Pathogen-associated molecular pattern (PAMP)-triggered immunity
QTL: Quantitative Trait Loci
R: A doubled haploid lines of the Strongfield/Blackbird population with transgressive FHB resistance
S: A doubled haploid lines of the Strongfield/Blackbird population with transgressive FHB susceptibility
SA: Salicylic acid
SF: *Triticum turgidum ssp. durum* cv. Srongfield
SNP: Single Nucleotide Polymorphism
UTR: Un-translated region
WGCNA: Weighted Gene Co-expression Network Analysis
