# Supplementary figures and images for: Weighted gene co-expression network analysis unveils gene networks associated with the Fusarium head blight resistance in tetraploid wheat
Source: BMC Genomics. 2019 Dec 3;20:925. doi: 10.1186/s12864-019-6161-8 (PMC6891979; doi:10.1186/s12864-019-6161-8)

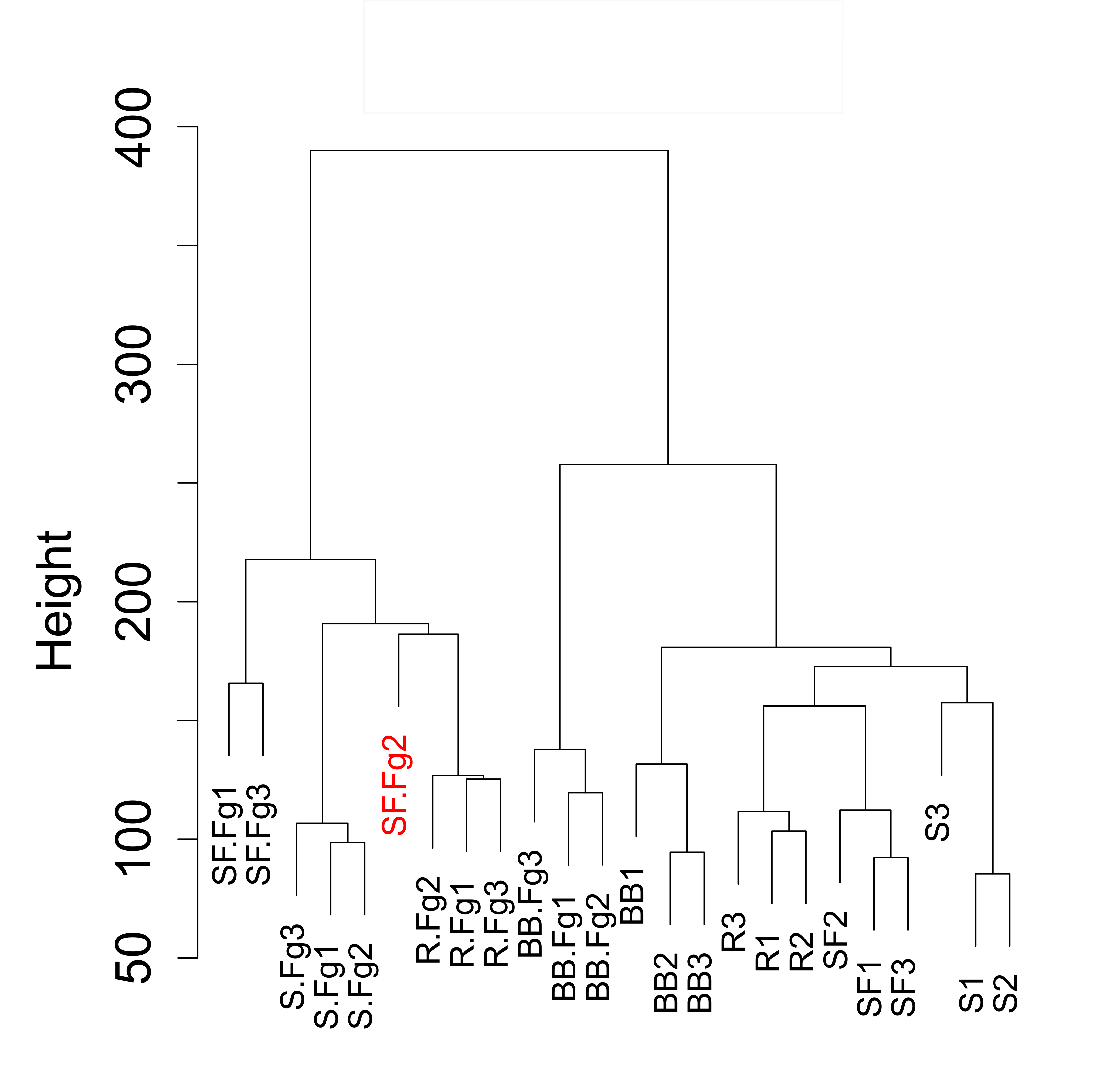

Supplement: Supplementary file 1 — Additional file 1. Hierarchical clustering of gene expression in samples used for weighted gene co-expression network analysis. Genotypes are durum wheat cv. Strongfield (SF), Triticum turgidum ssp. carthlicum line Blackbird (BB) and a resistant (R) and a susceptible (S) doubled haploid line of the SF/BB population. Samples that were inoculated with Fusarium graminearum have “.Fg” suffix. Samples are numbered sequentially to represent the three biological replicates per treatment. Marked in red is an outlier sample excluded from weighted gene co-expression network analysis. [file 12864_2019_6161_MOESM1_ESM.png]
