# Supplementary material for: Weighted gene co-expression network analysis unveils gene networks associated with the Fusarium head blight resistance in tetraploid wheat
Source: BMC Genomics. 2019 Dec 3;20:925. doi: 10.1186/s12864-019-6161-8 (PMC6891979; doi:10.1186/s12864-019-6161-8)
Supplement: Supplementary file 2 — Additional file 2. Primer pairs used for quantitative real time PCR of selected candidate hub genes. The candidate hub genes selected encode heat stress transcription factor A-2a (HSFA2A), putative late blight resistance R1C-3 (R1C-3), G-type lectin S-receptor-like serine/threonine-protein kinase SRK (SRK), heat shock cognate 70 kDa protein 2 (HSC-2) and serine/threonine-protein kinase PCRK1 (PCRK1). Expression data were normalized using α-tubulin as reference gene. Gene-IDs are from International Wheat Genome Sequencing Consortium (IWGSC) RefSeq v1.0 annotations. Genes belonging to various gene co-expression networks (modules) were tested. [file 12864_2019_6161_MOESM2_ESM.docx]

| Gene name | Gene ID | Primer sequence 5’-> 3’ | Module |
| --- | --- | --- | --- |
| *HSFA2A* | TraesCS5A01G533900 | F:TTGTTCTCCGAGCGGCAGCAGATA | Cyan |
|  |  | R:CCGCAGCTACCAGTGAAGGAACAA |  |
| *R1C-3* | TraesCS6D01G110100 | F:ATCGCATTGCTGAGCAGATGGAGA | FHB-M2 |
|  |  | R:GCAGCCTCCTTGTAGATTGCGGATA |  |
| *SRK* | TraesCS6B01G056200 | F:GAATCCTGCGGACTCAATTGGCACA | FHB-M4 |
|  |  | R:TTCGCAGGTTTCAAGTCAAGGTGGT |  |
| *HSC-2* | TraesCS1B01G294300 | F:ATTGGCAGGAGGTTCTCTGATCCC | Light yellow |
|  |  | R:CGCCCTTGTAGTTGACGACGATCAT |  |
| *PCRK1* | TraesCS2B01G555200 | F:TTTCCGGGACCTGAAGACGTCAAAC | FHB-M1 |
|  |  | R:CCACCAGCTTCACCAGATTGGGAT |  |
| *α-tubulin* | TraesCS4A02G065700 | F:GCCATCTACGACATCTGC | - |
|  |  | R:GGTCTGGAACTCGGTTATG |  |
